# Three cell deaths and a funeral: macrophage clearance of cells undergoing distinct modes of cell death

**DOI:** 10.1038/s41420-019-0146-x

**Published:** 2019-02-08

**Authors:** Katharina Klöditz, Bengt Fadeel

**Affiliations:** Division of Molecular Toxicology, Institute of Environmental Medicine, Karolinska Institutet, Stockholm, Sweden

## Abstract

Macrophage clearance of apoptotic cells has been extensively investigated, but less is known regarding the clearance of cells dying by other forms of programmed cell death, e.g., necroptosis or ferroptosis. Here, we established a model of three different cell deaths using the same cell line and the occurrence of distinct cell death modalities was verified by using the specific inhibitors, zVAD-fmk, necrostatin-1, and ferrostatin-1, respectively. Cell death was characterized by using transmission electron microscopy (TEM), the gold standard for the demarcation of different cell death modalities. Moreover, using annexin V as a probe, we could detect surface exposure of phosphatidylserine (PS) in all three types of cell death, and this was confirmed by using specific anti-PS antibodies. We then co-cultured the cells with human monocyte-derived macrophages and found that cells dying by all three death modalities were engulfed by macrophages. Macrophage clearance of apoptotic cells was more efficient when compared to necroptotic and ferroptotic cells with multiple internalized target cells per macrophage, as shown by TEM. We propose that clearance of dying cells also should be taken into account in the classification of different cell death modalities.

## Introduction

Cell death is a normal part of life. Cell death occurs during development and is required for tissue homeostasis in adult organisms. Several different forms of (programmed) cell death have been identified which can be distinguished by specific morphological features and/or corresponding biochemical processes (e.g., activation of specific kinases, proteases, and nucleases). Programmed cell clearance, in turn, is a conserved process of elimination of cell corpses^1,2^. However, it is not fully understood how phagocytes recognize and distinguish between different types of cell death.

Apoptosis was first described by Kerr et al.^3^ in 1972 and it is now well established that apoptosis plays an important role in health and disease^4^. Two major apoptotic pathways are described in mammalian cells: the so-called extrinsic and intrinsic pathways. The former pathway is triggered by binding of a ligand to a cell death receptor expressed on the plasma membrane leading to oligomerization and intracellular assembly of a death-inducing signaling complex (DISC) with subsequent caspase activation. The death receptor-mediated pathway is important for apoptosis in the immune system^5^. The intrinsic or mitochondria-mediated apoptotic pathway is characterized by mitochondrial outer membrane permeabilization leading to the release of pro-apoptotic mitochondrial proteins including cytochrome c and apoptosis-inducing factor (AIF) into the cytosol. The formation of a complex, referred to as the apoptosome, between cytochrome c, apoptotic protease-activating factor-1 (Apaf-1), and pro-caspase-9 leads to caspase activation and apoptosis^6^. The intrinsic apoptosis pathway is widely conserved through evolution, from worms to humans^7,8^. In 2005, Yuan and co-workers described a novel, non-apoptotic, cell death mechanism termed necroptosis that is regulated by receptor-interacting serine/threonine kinases 1 and 3 (RIPK1/3)^9^. Necrostatin-1 was identified as a specific inhibitor of necroptosis. Subsequent studies have implicated the mixed lineage kinase domain like pseudokinase (MLKL) as a key mediator of necrosis signaling downstream of RIP3^10^. Fas-associated death domain (FADD) is part of the DISC and acts as an adaptor for pro-caspase-8. The accumulation and oligomerization of pro-caspase-8 facilitate its activation and result in the activation of downstream effector caspases^5^. Cells expressing dominant negative FADD (FADD-DN) lacking the death effector domain (DED) fail to activate caspase-8 and do not undergo apoptosis. Instead, incubation with TNF-α was shown to trigger necroptosis likely via the binding of FADD to RIPK1 and RIPK3 in a so-called necroptosome complex^11^. Ferroptosis is a more recently discovered form of non-apoptotic cell death characterized by a lethal, iron-dependent accumulation of lipid hydroperoxides^12^. Stockwell and co-workers showed that glutathione peroxidase 4 (GPX4) is a key regulator of ferroptosis, and ferrostatin-1 was identified as an inhibitor of ferroptosis^12^. Necroptosis and ferroptosis are implicated in various pathological conditions^12,13^.

Cell death plays an important role in inflammation^14^. However, it is overly simplified to say that necrosis triggers inflammation while apoptosis resolves inflammation. Cell death, and the clearance of dying cells by macrophages and other phagocytic cells, also plays a regulatory role in inflammation^15,16^. Moreover, it is pertinent to note that cell death signaling molecules also have non-lethal roles in inflammation^14^. For instance, caspase-8 blocks RIPK3-mediated activation of the NLRP3 inflammasome^17^. Indeed, it has been speculated that programmed necrosis may not be the cause but may well result as a consequence of inflammation^18^. Phagocytosis of apoptotic cells has been investigated in considerable detail and it is generally believed that phagocytes distinguish apoptotic cells from healthy cells via specific engulfment receptors, which recognize “eat-me” signals on the surface of the dying cell^19^. The best-studied “eat-me” signal is the exposure of the anionic phospholipid phosphatidylserine (PS), an evolutionarily conserved signal from nematodes to humans. However, cells may undergo apoptosis in the absence of PS exposure^20^ and macrophage engulfment of cells triggered to undergo death receptor-mediated apoptosis may occur prior to the externalization of PS on the target cells^21^. Furthermore, PS exposure has been documented in cells dying by necrosis^22,23^. Thus, while PS exposure (as determined by labeling of the cells with the PS-binding protein, annexin V) is often considered as a marker of apoptotic cell death, PS exposure cannot be considered a specific ligand for the recognition of either apoptotic or necrotic cells^24^ and other signals are likely to confer “meaning” to cell clearance. It remains unknown whether ferroptotic cells externalize PS prior to their disintegration. The aim of this study was to investigate macrophage clearance of cells dying by three different modes of cell death, i.e., apoptosis, necroptosis, and ferroptosis, and to determine whether PS exposure occurred in cells undergoing different modes of cell death.

## Results

### Establishment of a cell model to study three different cell deaths

**Fig. 1:**
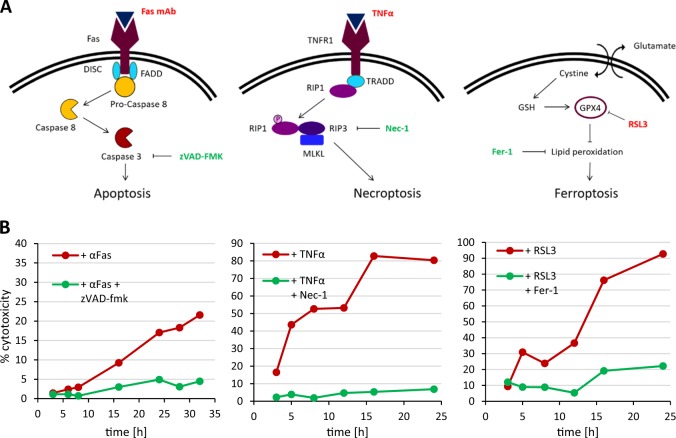
Three modes of programmed cell death. (**a**) Schematic figures showing the cell death pathway for apoptosis, necroptosis, and ferroptosis. The cell death inducers used in the present study are indicated in red and the specific inhibitors are shown in green. (**b**) Time course of cell death as evidenced by LDH release in Jurkat (apoptosis, ferroptosis) or FADD-DN Jurkat cells (necroptosis). Cell death was induced by the addition of 250 ng/mL Fas antibody, 10 ng/mL TNF-α, or 2 µM RSL3 in the presence or absence of the respective cell death inhibitors, zVAD-fmk (10 µM), Nec-1 (40 µM), or Fer-1 (5 µM). Refer to Figure S1–S3 for additional morphological and biochemical indices of cell death, and Figure S4 for cell death results based on light scatter

We chose the human T cell line Jurkat as a model to study apoptosis, necroptosis, and ferroptosis (Fig. 1a). Jurkat cells have been widely employed as a model to study Fas-mediated apoptosis^25^ while FADD-DN Jurkat cells serve as a model to study TNF-α-induced necroptosis^26^. The GPX4 inhibitor, RSL3 has been shown to trigger ferroptosis in different cell types^27^ and this compound was used here to trigger ferroptotic cell death in Jurkat cells. We noted in preliminary experiments that cell density was an important determinant of sensitivity to ferroptotic cell death, but optimization of the cell culture conditions enabled us to trigger apoptosis, necroptosis, and ferroptosis in Jurkat cells using 250 ng/mL Fas antibody, 10 ng/mL TNF-α, and 2 µM RSL3, respectively (Fig. 1b). Apoptosis was blocked by the pan-caspase inhibitor, zVAD-fmk (10 µM), and necroptosis was inhibited by the RIPK1 inhibitor, Nec-1 (40 nM), while ferroptosis was prevented by adding the lipid antioxidant, Fer-1 (5 µM). The fact that lactate dehydrogenase (LDH) release was less pronounced in the case of apoptosis confirms that this mode of cell death transpires without significant loss of membrane integrity^3^.

To further confirm the induction of different cell death modalities, we examined biochemical characteristics known to accompany each of these cell deaths. We also performed transmission electron microscopy (TEM) analysis in order to elucidate the ultrastructural features of cell death. As shown in Figure S1, Fas ligation resulted in the activation of caspase-3-like enzymes. Furthermore, at 24 h, the cells displayed condensed or in some cases crescent-shaped chromatin and fragmented nuclei, and a high degree of intracellular vesicles indicative of the fragmentation of intracellular membrane structures. In TNF-α-treated FADD-DN Jurkat cells, we noted phosphorylation of MLKL and this was blocked by Nec-1 (Figure S2). TEM analysis at 24 h showed that these cells lacked typical apoptotic features such as chromatin condensation. Instead, necroptotic cells were characterized by organelle swelling and disrupted mitochondrial cristae. Moreover, we observed mitochondrial membrane rupture as well as electron dense lipid droplets. Finally, in cells treated with RSL3, time-dependent lipid peroxidation was observed and this was blocked by Fer-1 (Figure S3). Ferroptotic cells also showed altered mitochondrial morphology and mitochondrial membrane rupture was commonly observed at 24 h, as revealed by TEM analysis. No nuclear condensation was noted in ferroptotic cells, but the shape of the cell nuclei appeared less rounded when compared to untreated control cells. We observed numerous lipid droplets in ferroptotic cells. Flow cytometric analysis of forward/side scatter suggested a comparable degree of cell death at 24 h for cells undergoing apoptosis, necroptosis, and ferroptosis (Figure S4). Similar results were obtained by vital dye exclusion using Trypan blue (data not shown).

### PS exposure occurs in all three forms of programmed cell death

**Fig. 2:**
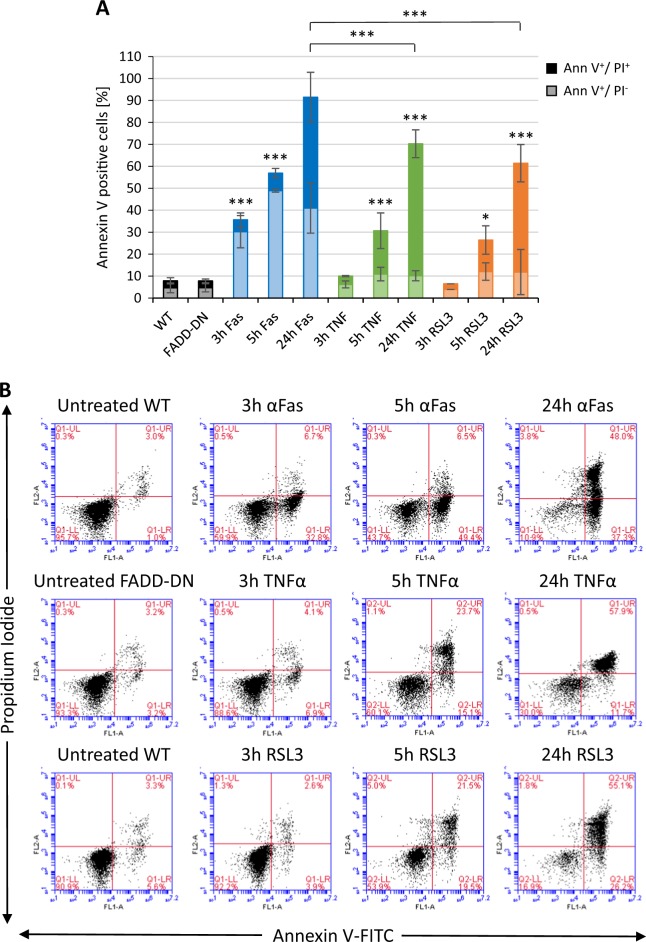
PS exposure in programmed cell death. **a** Quantification of annexin V-FITC/PI staining of Jurkat cells or FADD-DN Jurkat cells exposed to 250 ng/mL Fas antibody, 10 ng/mL TNF-α, or 2 µM RSL3 for the indicated time-points. The lower bars (light shading) represent the annexin V-positive/PI-negative cells and the upper bars (dark shading) the annexin V-positive/PI-positive (“double-positive”) cells. Data shown are mean values ± S.D. (*n* = 3). Statistically significant differences were noted for all the indicated samples versus control, and for the 24 h apoptotic samples versus the 24 h necroptotic and ferroptotic samples. *p < 0.05, ***p < 0.001. **b** Representative annexin V-FITC/PI results for cells undergoing apoptosis, necroptosis, and ferroptosis. The percentages of cells in each quadrant are shown. Refer to Figure S5 for annexin V-FITC results with/without cell death inhibitors versus staining with anti-PS antibodies, and Figure S6 for results on the (loss of) CD31 expression following cell death induction

Next, we asked whether PS exposure occurs in all three forms of cell death. To this end, cell death was induced as described above and cells were then stained with FITC-labeled annexin V and propidium iodide (PI). Annexin V binds to negatively charged phospholipids such as PS in a calcium-dependent manner and is commonly used to study PS exposure. PI is a cell-impermeable DNA intercalating dye used to detect cells that have lost their membrane integrity. We confirmed the occurrence of an annexin V-positive cell population for all three forms of cell death. Fas-triggered (apoptotic) Jurkat cells showed the highest amount of annexin V-positive cells at 24 h (Fig. 2a). Importantly, PS exposure was observed also at early time-points (3–5 h) and this was most pronounced for cells undergoing apoptosis, but PS exposure was also noted for necroptotic and ferroptotic cells prior to the loss of membrane integrity, as determined by PI staining (Fig. 2b). Furthermore, PS exposure was blocked by zVAD-fmk, Nec-1, and Fer-1, respectively (Figure S5A). However, while annexin V binds to PS, it can also bind other lipids including cardiolipin^26^. To confirm that PS exposure occurred, we also stained cells using PS-specific antibodies (Figure S5B). PS exposure was noted for all three cell death modalities, but the number of PS-positive apoptotic cells detected by PS antibodies was lower than expected based on the annexin V results. Fas-triggered cells are known to expose both non-oxidized and oxidized PS^28^ and it remains possible that these antibodies bind poorly to oxidized PS. Phagocytosis of dying cells is facilitated by the exposure of “eat-me” signals, but previous studies have also suggested that a reduction of the expression of repellant signals such as CD31 may also play a role in programmed cell clearance^29^. We therefore examined whether cells undergoing apoptosis, necroptosis, or ferroptosis displayed a loss of expression of CD31. We observed a minor reduction in CD31 expression in apoptotic cells, but not in necroptotic or ferroptotic cells (Figure S6A-C).

### Macrophages engulf cells dying according to different modalities

**Fig. 3:**
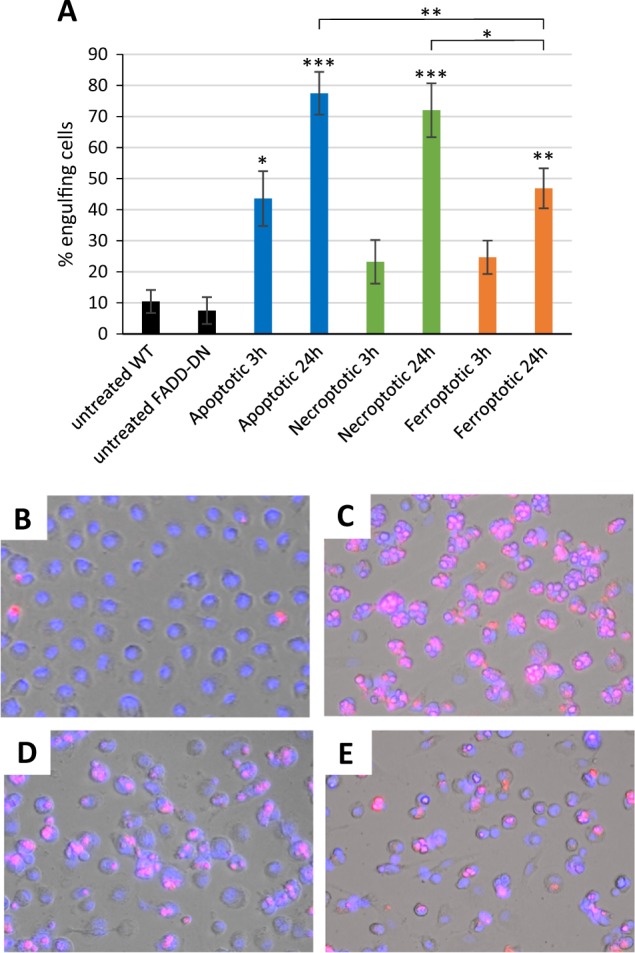
Macrophage engulfment of dying cells. **a** Quantification of macrophage engulfment of target cells triggered to undergo apoptosis, necroptosis, or ferroptosis for 3 or 24 h. Macrophages and target cells were co-cultured for 1 h and results were scored as described in Materials and methods. Data shown are mean values ± S.D. (*n* = 3). Statistically significant differences were noted for the indicated samples versus the respective control (i.e., untreated WT or untreated FADD-DN cells), and for the apoptotic samples versus the necroptotic and ferroptotic samples at 24 h. *p < 0.05, **p < 0.01, ***p < 0.001. Below are representative images of control macrophages (**b**), or macrophages engaged in the ingestion of TAMRA-labeled apoptotic (**c**), necroptotic (**d**), or ferroptotic (**e**) target cells treated for 24 h with 250 ng/mL Fas antibody, 10 ng/mL TNF-α, or 2 µM RSL3, respectively, prior to macrophage co-culture for 1 h

**Fig. 4:**
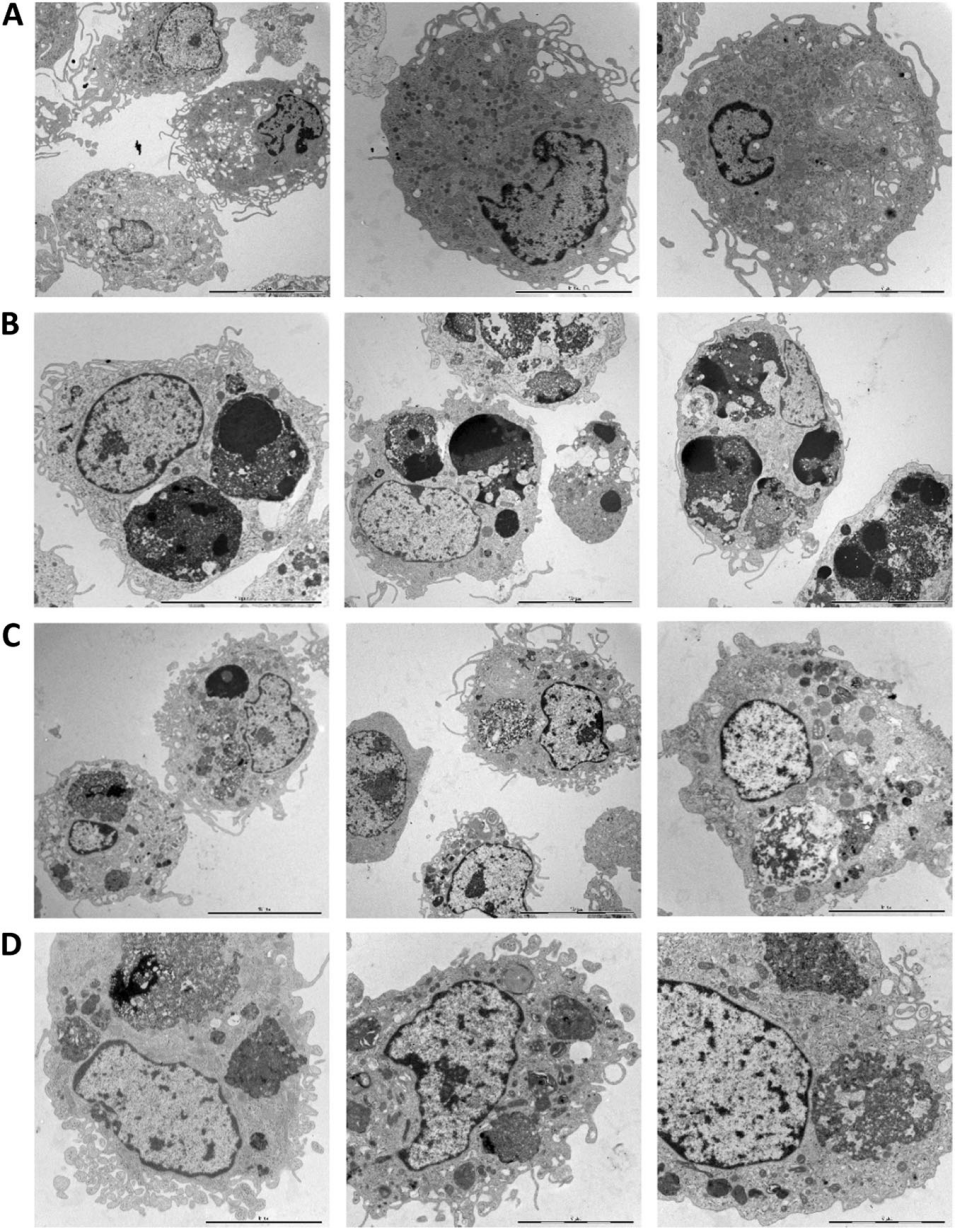
Macrophage engulfment of dying cells. Jurkat cells or FADD-DN Jurkat cells were triggered to undergo three different cell death modalities for 24 h using anti-Fas antibodies, TNF-α, and RSL3, respectively, and were then co-cultured for 1 h with primary human monocyte-derived macrophages. Non-engulfed cells were washed away and macrophages were collected, fixed, and processed for TEM. Shown here are images of control macrophages (**a**) and macrophages engulfing apoptotic (**b**), necroptotic (**c**), or ferroptotic (**d**) Jurkat cells. Scale bars represent 5, 10, or 20 µm. Note the presence of multiple, largely intact target cells with condensed chromatin in macrophages in (**b**) as opposed to the uptake of severely fragmented cells in (**c**) and (**d**)

Cell clearance defines the “meaning” of cell death^15^. We therefore asked whether apoptotic, necroptotic, and ferroptotic target cells are recognized by macrophages and whether they are cleared with the same efficiency. To this end, we used human monocyte-derived macrophages (HMDMs), professional phagocytes that are far more proficient at engulfing cells than, for instance, macrophage-like THP-1 or RAW264.7 cell lines. Target cells were TAMRA-labeled and co-cultured with HMDMs for 1 h, and phagocytosis efficiency was quantified by using fluorescent microscopy techniques (Fig. 3a). Several notable observations could be made: (1) cells undergoing apoptosis were already engulfed to a significant extent after 3 h of Fas ligation; (2) cells undergoing apoptosis were more readily engulfed after 24 h of cell death induction when compared to necroptotic and ferroptotic cells; (3) macrophages appeared to engulf several apoptotic target cells per macrophage (refer to Fig. 3c versus 3d and 3e). To confirm that macrophages had ingested their prey as opposed to the attachment of target cells to the surface of macrophages, we performed TEM analysis after 1 h of co-culture. The results shown in Fig. 4 provide conclusive evidence of macrophage engulfment of cells undergoing apoptosis, necroptosis, and ferroptosis, and it is also noted that while apoptotic cells appeared to be internalized as intact cells, the necroptotic and ferroptotic cells were internalized to a larger extent as fragmented cells. TEM also confirmed that multiple apoptotic cells were ingested per macrophage.

## Discussion

Several different cell death modalities and their molecular underpinnings have been described in recent years. However, our knowledge regarding the clearance of cells undergoing different types of cell death as well as how these cell deaths are decoded by the immune system remains limited. In addition, despite early evidence that PS exposure is not a specific marker of apoptosis, it is still fairly common to use annexin V positivity as a proxy for apoptotic cell death. To begin to shed some light on these issues, we have established a model that allows for the investigation of three different forms of programmed cell death (apoptosis, necroptosis, and ferroptosis) in a single cell type. Furthermore, we used primary HMDMs to monitor phagocytosis, and it should be noted that the latter assay (the “funeral”) was performed with the same ratio of target cells to phagocytes for all three modes of cell death.

We believe that morphological changes coupled with specific molecular features are required to make a differential diagnosis of cell death, and we dismiss the notion that there is a single biochemical feature with which to distinguish different modes of cell death. In the present study, we triggered cell death using agonistic anti-Fas antibodies, or TNF-α, or the GPX4 inhibitor, RSL3, and we could show that cell death in each instance could be blocked by the cognate inhibitor, i.e., the caspase inhibitor, zVAD-fmk for apoptosis, the RIPK1 inhibitor, Nec-1 for necroptosis, and the lipid antioxidant, Fer-1 for ferroptosis. We demonstrated that these cell deaths displayed distinct morphological changes. We also noted that cell surface exposure of PS occurred in all three cases as evidenced by using annexin V as well as specific anti-PS antibodies, though we observed that the population of “single-positive” cells (i.e., annexin V-positive and PI-negative) was significantly elevated only in the case of apoptosis. These observations are noteworthy as this suggests that PS exposure is not an apoptosis-specific signal, and PS exposure is not necessarily caspase-dependent, though it may very well be caspase-dependent in cells undergoing apoptotic cell death^30,31^. In necroptotic cells, PS exposure is caspase-independent^26^ and transpires downstream of MLKL^32,33^. PS exposure was suggested not to occur in GPX4-deficient fibroblasts^34^, but we show here that some PS exposure occurs in Jurkat cells treated with RSL3, an inhibitor of GPX4, suggesting cell type-specific differences.

The present observations certainly do not rule out a role for PS exposure in cell clearance. On the contrary, the importance of PS-mediated phagocytosis is underscored by the spectrum of inflammatory and autoimmune disorders caused by defects in PS receptors and related signaling molecules^35^. Furthermore, PS is a conserved signal for phagocytic uptake of dying cells^36^. Notably, our present results show that apoptotic cells exhibited the highest degree of PS exposure (at 24 h) and apoptotic cells were more efficiently engulfed than necroptotic and ferroptotic cells, suggesting a correlation between PS exposure and cell clearance. We also found that CD31 expression was reduced at 24 h and completely lost in Fas-triggered cells at 48 h (not shown), but it is unclear to what extent the loss of CD31 contributed to cell clearance. Our study demonstrated that macrophages are capable of ingesting several apoptotic target cells at once, indicating that apoptotic cells are especially appetizing for macrophages, while necroptotic and ferroptotic cells were more frequently internalized as individual cells or cell fragments. It remains to be understood how clearance of apoptotic, necroptotic, and ferroptotic cells will influence subsequent immune responses in health or disease^37,38^. PS exposure is likely to be only one of many elements of the complex signaling “code” that dying cells transmit to their environment^19,39^. Furthermore, in addition to different cell death modalities, there is also considerable heterogeneity among macrophages and other phagocytes^40^. To conclude, we suggest that cell clearance—the final stage of the cell death process—should be taken into account in the classification of different modes of cell death^2^.

## Materials and methods

### Jurkat cell culture and cell death

The human leukemic Jurkat T cell line (Sigma Aldrich) or FADD-dominant negative (DN) Jurkat cells^41^ (kindly provided by Dr. John Blenis, Harvard Medical School) were cultured in RPMI-1640 medium (Sigma-Aldrich) supplemented with 10% heat-inactivated fetal bovine serum (FBS), 2 mM L-glutamine, 100 U/mL penicillin, and 100 µg/mL streptomycin (Thermo Fisher Scientific). Cell death was induced in Jurkat cells or FADD-DN Jurkat cells seeded at a density of 0.5 × 10^6^ cells/mL by adding 250 ng/mL anti-Fas monoclonal antibody (MBL International), 10 ng/mL TNF-α (Thermo Fisher Scientific), or 2 µM (1S,3R)-RSL3 (Selleck Chemicals), respectively. We used Jurkat cells as a model to induce both apoptosis and ferroptosis while FADD-DN Jurkat cells served as a model to induce necroptosis, as shown before^26^. Inhibition of apoptosis was achieved by addition of the caspase inhibitor, zVAD-fmk (10 µM) (Sigma Aldrich), while necroptosis was blocked by RIPK1 inhibitor, necrostatin-1 (Nec-1) (40 µM) (Sigma Aldrich), and ferroptosis was inhibited by lipid antioxidant, ferrostatin-1 (Fer-1) (5 µM) (Sigma Aldrich). The inhibitors were added 1 h prior to the cell death inducers.

### Transmission electron microscopy

For ultrastructural analysis using TEM, Jurkat cells were triggered to undergo cell death for 24 h. Additionally, macrophages co-incubated with dying cells for 1 h were also processed for TEM. Cells were fixed in 2.5% glutaraldehyde in 0.1 M phosphate buffer, pH 7.4 at room temperature for 30 min and further fixed overnight in the refrigerator. The samples were rinsed in 0.1 M phosphate buffer and centrifuged prior to post-fixation using 2% osmium tetroxide in 0.1 M phosphate buffer, pH 7.4 at 4 °C for 2 h. Following the post-fixation, the cells were step-wise dehydrated in ethanol followed by acetone and LX-112 infiltration and finally embedded in LX-112. Ultrathin sections (approx. 50–60 nm) were prepared using a Leica EM UC6, contrasted with uranyl acetate followed by lead citrate, and examined in Hitachi HT 7700 electron microscope (Hitachi High-Technologies). Digital images were acquired using a 2kx2k Veleta CCD camera (Olympus Soft Imaging Solutions).

### Phagocytosis assay

HMDMs were obtained from peripheral blood mononuclear cells as described previously^42^. Briefly, cells were isolated from buffy coats of healthy blood donors by using a Lymphoprep™ density gradient. Buffy coats were purchased from the Karolinska University Hospital blood bank and all samples were completely anonymized prior to handling in the laboratory. CD14-positive mononuclear cells were then isolated by using CD14 magnetic MicroBeads (Miltenyi Biotec) and cultured in RPMI-1640 medium supplemented with 10% heat-inactivated FBS, 2 mM glutamine, 100 U/mL penicillin, and 100 μg/mL streptomycin (Thermo Fisher Scientific) and 50 ng/mL recombinant human macrophage colony-stimulating factor (M-CSF) (PeproTech) for 4 days to induce differentiation into macrophages. Cell death was induced in target cells as described and the cells were then re-suspended in fresh culture medium without FBS and stained for 30 min with 50 µg/mL 5(6)-carboxytetramethylrhodamine *N*-hydroxysuccinimide ester (TAMRA) (Sigma Aldrich). Target cells were then re-suspended in complete RPMI-1640 medium and co-cultured with the HMDMs for 1 h. The same ratio of phagocytes to target cells (1:5) was used for all samples. Following co-culture, extensive washing using ice-cold 0.5 mM EDTA in PBS was performed to remove non-engulfed target cells and HMDMs were then fixed in 4% formaldehyde in PBS. Cell nuclei were counterstained with Hoechst 33342 (Sigma Aldrich) and engulfment efficiency was investigated and quantified using an inverted Nikon ECLIPSE TE2000-S fluorescence microscope. To this end, six visual fields each containing approx. 300 macrophages were captured for each condition. The total number of macrophages and the number of macrophages positive for uptake of TAMRA-labeled target cells were counted and the percentage of phagocytosis-positive macrophages was calculated. The quantification is based on three independent experiments using HMDMs isolated from three different human donors.

### Cytotoxicity assessment

The LDH Cytotoxicity Assay Kit (Thermo Fisher Scientific) was used to determine LDH release into the cell culture medium according to the manufacturer’s instructions. In brief, cells were suspended at a density of 0.5 × 10^6^ cells/mL in 24-well plates and cell death was induced as indicated. Samples lysed in lysis buffer (provided in the kit) at 37 °C for 45 min were used to determine maximum LDH release. Untreated samples represent spontaneous LDH release. LDH release was measured by determining absorbance at 492 and 630 nm using the Tecan Infinite^®^ F200 plate reader. To complement this assay, cell viability/cell death was also monitored on the basis of changes in forward scatter and side scatter as determined by flow cytometry (refer to “PS and CD31 exposure” section), and by vital dye exclusion. To this end, cell suspensions were mixed with 0.4% Trypan Blue Dye solution (Bio-Rad Laboratories) and cell counting was performed using the TC20™ Automated Cell Counter (Bio-Rad Laboratories).

### Caspase activity

Caspase 3-like activity was investigated by measuring cleavage of the fluorogenic peptide DEVD-AMC (aspartate-glutamate-valine-aspartate-7-amino-4-methyl-coumarin). Briefly, Jurkat cells were pelleted and frozen on microtitre plates at 0.25 × 10^6^ cells in 25 µL. The substrate (50 µM) was dissolved in a standard reaction buffer [100 mM HEPES, 10% sucrose, 5 mM dithiothreitol (DTT) and 0.1% CHAPS, 0.0001% NP-40, pH 7.25] and 50 µL was added to each well. Enzyme-catalyzed cleavage of AMC was followed by measurement of the fluorescent intensity every 5 min for a 40 min period in a Tecan plate reader at 360 nm excitation and 465 nm emission wavelengths.

### Lipid peroxidation

Lipid peroxidation was determined after incubation of cells with RSL3 at the indicated time-points. Cells were collected and resuspended in PBS containing 2 µM of the oxidation-sensitive fluorescent sensor, BODIPY^®^ 581/591 (Thermo Fisher Scientific). Following incubation for 30 min in the dark, analysis was performed using the BD Accuri™ C6 flow cytometer operating with BD Accuri™ C6 software (BD Biosciences).

### PS and CD31 exposure

The Annexin V-FITC Apoptosis Detection Kit was applied according to the manufacturer’s instructions (Merck). Briefly, Jurkat cells were collected following cell death induction and resuspended in Annexin V staining buffer for 30 min. PI (final concentration: 0.6 µg/mL) was added to each sample prior to analysis. Samples were analyzed using the BD Accuri™ C6 flow cytometer operating with BD Accuri™ C6 software. Additionally, flow cytometric analysis was performed on cells stained with antibodies against PS. To this end, cells were resuspended in PBS and stained with 0.2 µg/mL anti-PS-Alexa Fluor 488 antibody or the isotope control antibody, mouse IgG Alexa Fluor 488 (both from Merck Millipore) for 30 min. Flow cytometry was performed using the BD Accuri™ C6 flow cytometer and BD Accuri™ C6 software. Surface expression of CD31 was also determined by using flow cytometry. Following cell death induction, cells were stained with 1 µg/mL of the mouse anti-human CD31-FITC antibody (Bio-Rad Laboratories) or the FITC-labeled IgG1 isotope control antibody (BD Biosciences) and analyzed on a BD Accuri™ C6 flow cytometer.

### Western blotting

For western blotting, cells were collected and lysed overnight at 4 °C in RIPA buffer [50 mM Tris HCl (pH 7.4), 150 mM NaCl, 1% Triton X-100, 0.25% sodium deoxycholate, 0.1% SDS, 1 mM EDTA]. Protease- and phosphatase inhibitors (Mini EDTA-free Protease Inhibitor Cocktail, Sigma Aldrich; 1 mM PMSF, Thermo Fisher; PhosSTOP, Sigma Aldrich) as well as 1 mM DTT (Sigma Aldrich) were freshly added to the RIPA buffer. 60 µg total protein were loaded into each well of a NuPAGE 4–12% Bis–Tris gradient gel (Thermo Fisher) and subjected to electrophoretic separation of the proteins. The proteins were then transferred to a Hybond Low-fluorescent 0.2 µm PVDF membrane (Amersham), blocked for 1 h in Odyssey^®^ Blocking Buffer (PBS) (LI-COR), and stained overnight at 4 °C with antibodies against phospho-MLKL (Abcam, ab187091), with GAPDH (Thermo Fisher) as a loading control. The goat anti-rabbit IgG (H+L) HRP-conjugated antibody (Thermo Fisher Scientific) or the goat anti-mouse IRDye 680RD antibody (LI-COR Biotechnology GmbH) were used as secondary antibodies. The proteins were detected and analyzed using the Kodak medical X-ray processor using Clarity ECL western blotting substrates (BioRad) and Super RX-N film (Fuji), or the LI-COR Odyssey^®^ CLx scanner using Odyssey^®^ Image Studio software.

### Statistical analysis

The results reported in the graphs represent average values from at least 3 independent experiments ± S.D. Statistical analysis was performed using GraphPad Prism 5.02 software. One-way analysis of variance (ANOVA) followed by Bonferroni’s multiple comparison test was used to investigate differences between samples. *p* < 0.05 was considered statistically significant. *p < 0.05, **p < 0.01, ***p < 0.001.

## Supplementary information

Figure S1-S6

Supplemental Material File #1
