# Supplementary material for: Three cell deaths and a funeral: macrophage clearance of cells undergoing distinct modes of cell death
Source: Cell Death Discov. 2019 Feb 8;5:65. doi: 10.1038/s41420-019-0146-x (PMC6368547; doi:10.1038/s41420-019-0146-x)
Supplement: Supplementary file 1 — Figure S1-S6 [file 41420_2019_146_MOESM1_ESM.pptx]

## Slide 1
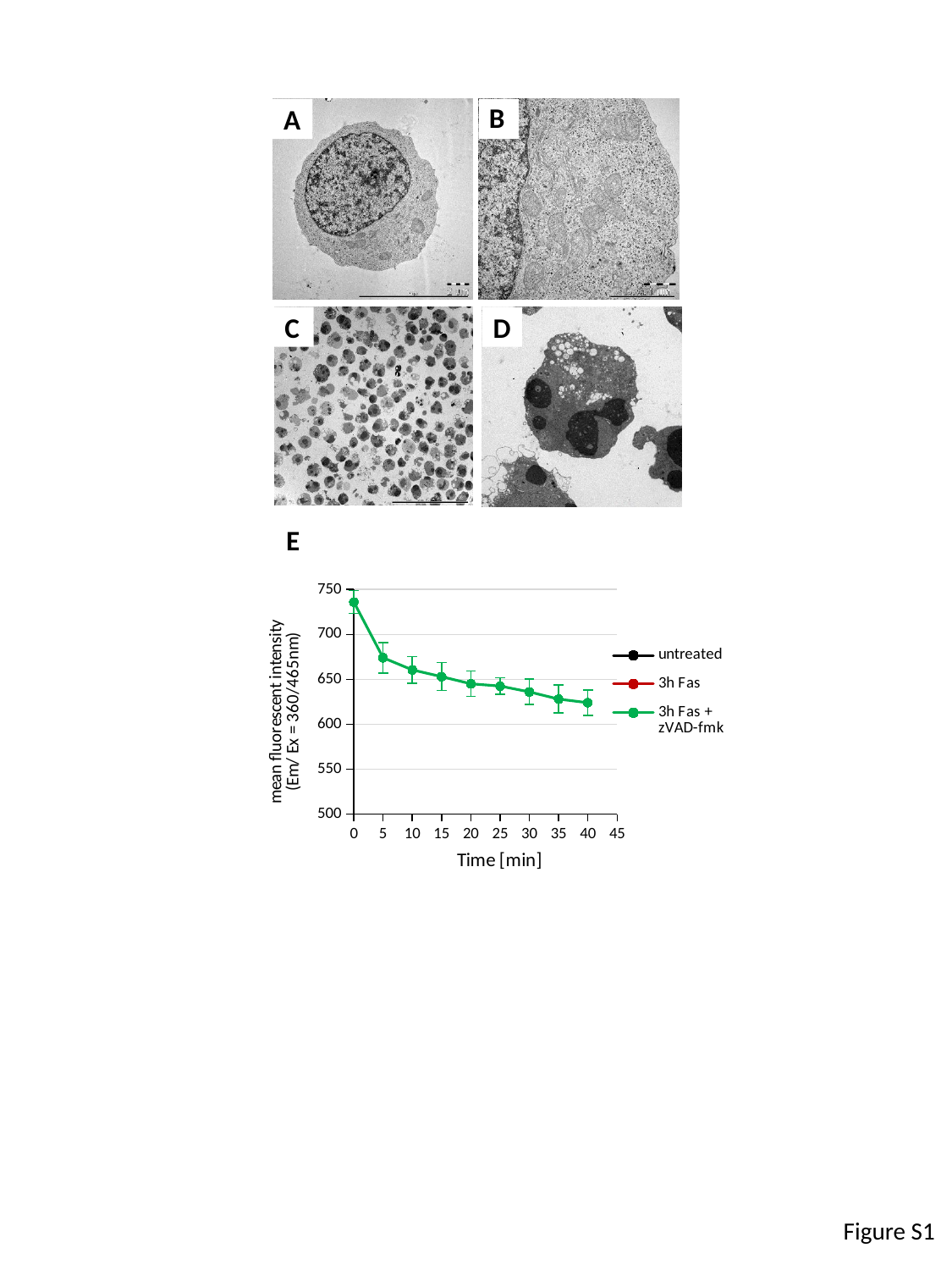

B
A
D
C
E
### Chart
| Category | untreated | 3h Fas | 3h Fas + zVAD-fmk |
|---|---|---|---|Figure S1

## Slide 2
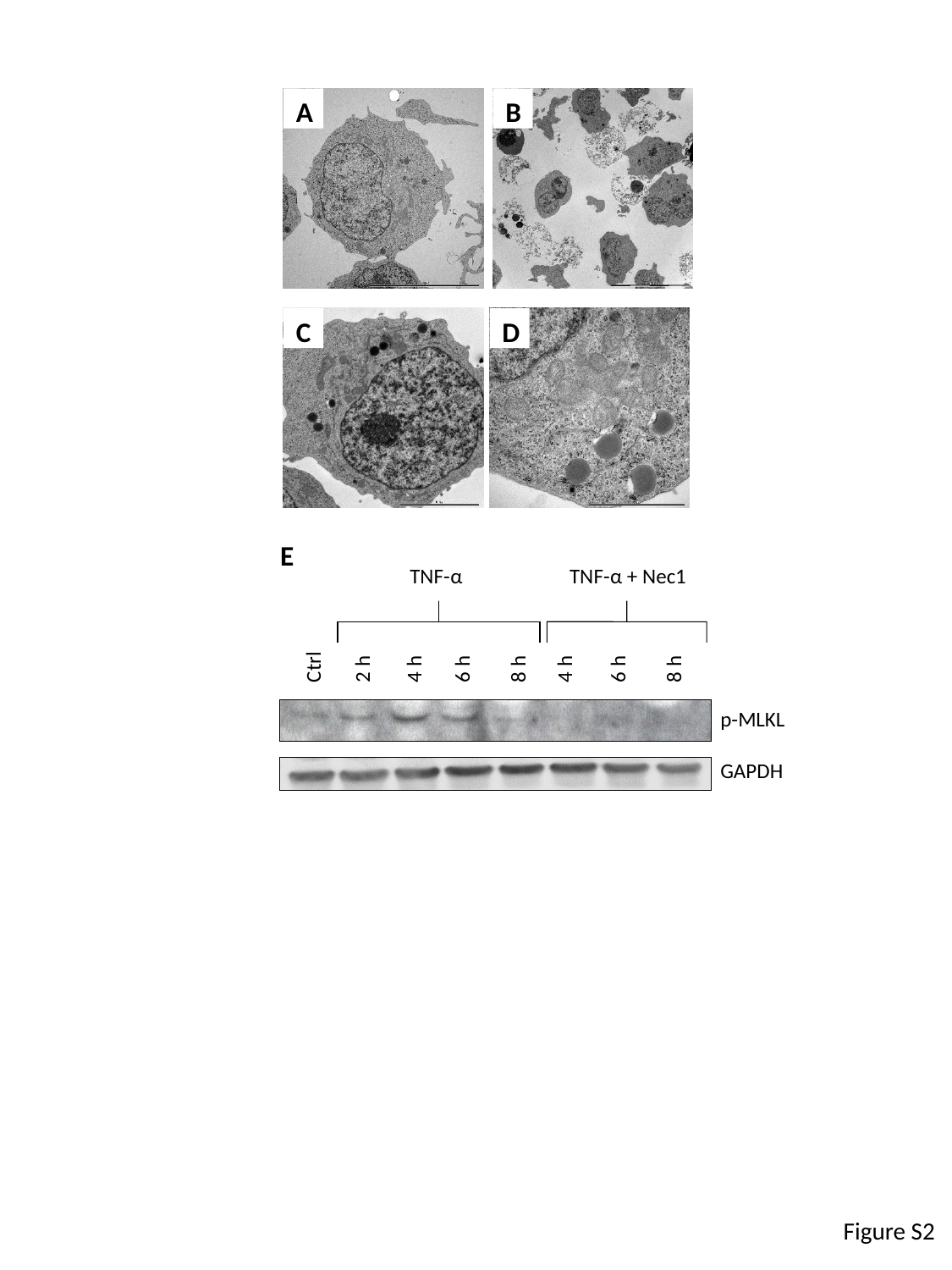

A
B
C
D
E
TNF-α
TNF-α + Nec1
Ctrl
2 h
4 h
6 h
8 h
4 h
6 h
8 h
p-MLKL
GAPDH
Figure S2

## Slide 3
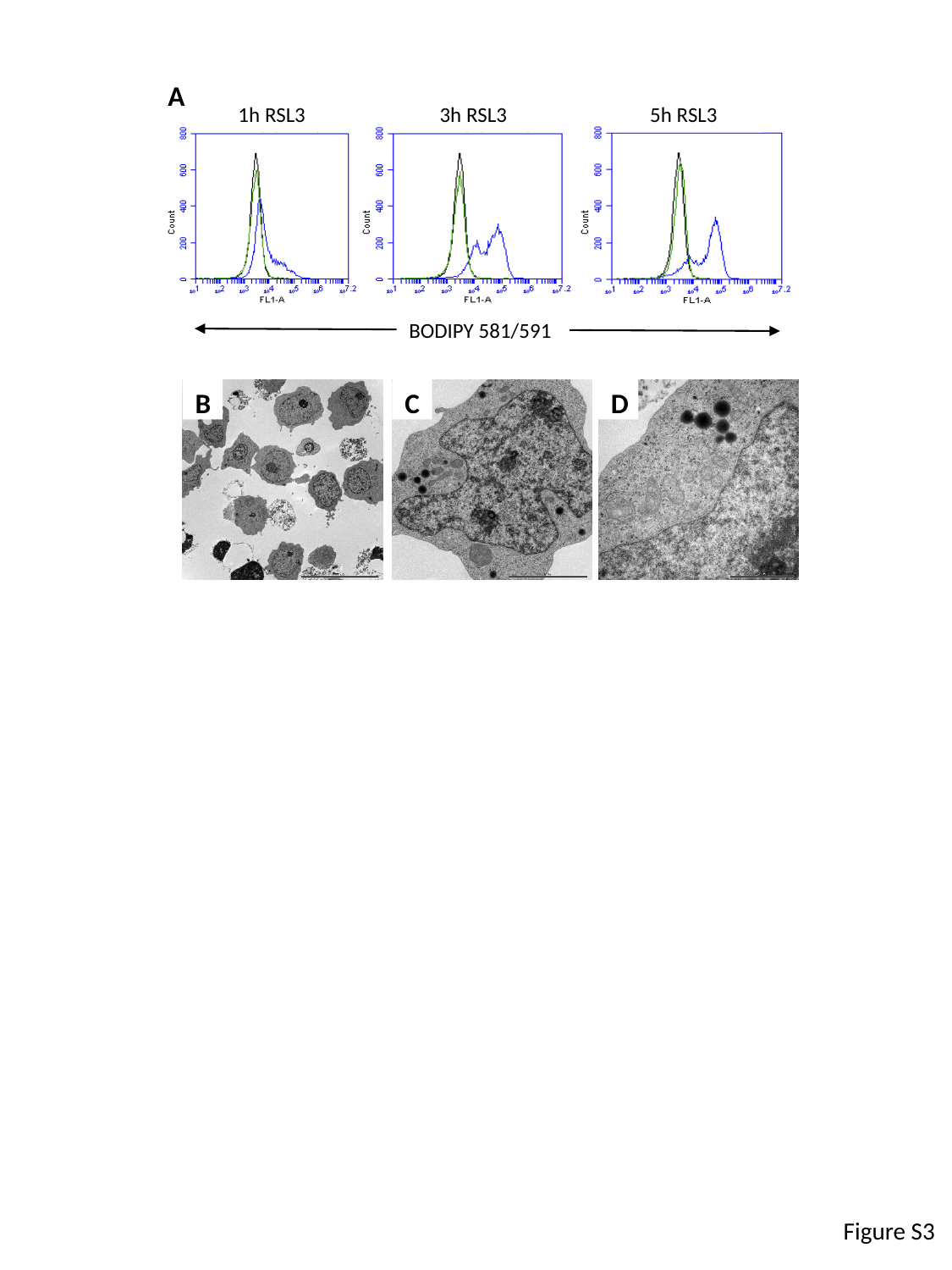

A
5h RSL3
1h RSL3
3h RSL3
BODIPY 581/591
B
C
D
Figure S3

## Slide 4
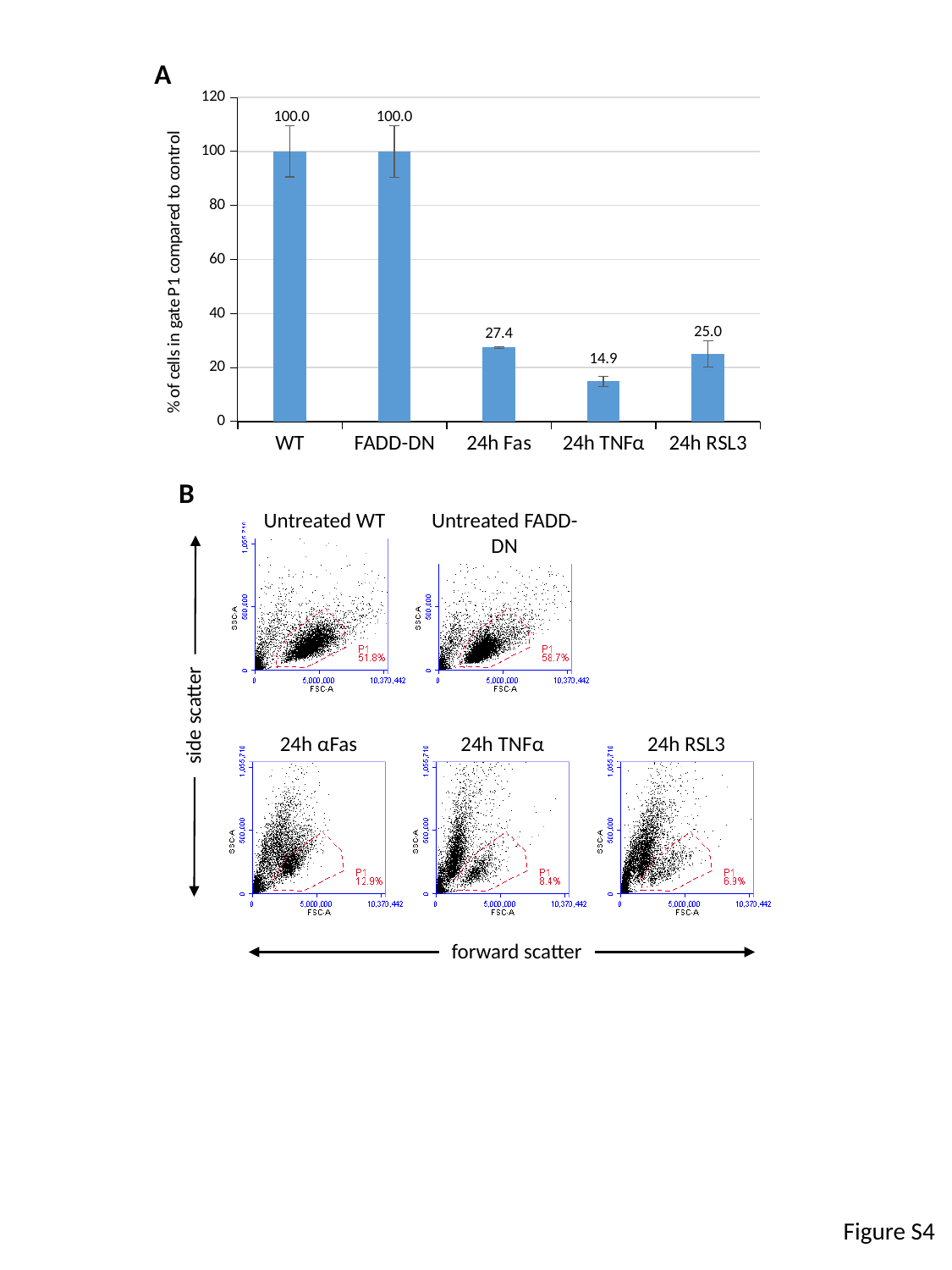

A
### Chart
| Category | |
|---|---|
| WT | 100.0 |
| FADD-DN | 100.0 |
| 24h Fas | 27.39432825356043 |
| 24h TNFα | 14.869010967104389 |
| 24h RSL3 | 25.00638794971519 |B
Untreated WT
Untreated FADD-DN
side scatter
24h αFas
24h TNFα
24h RSL3
forward scatter
Figure S4

## Slide 5
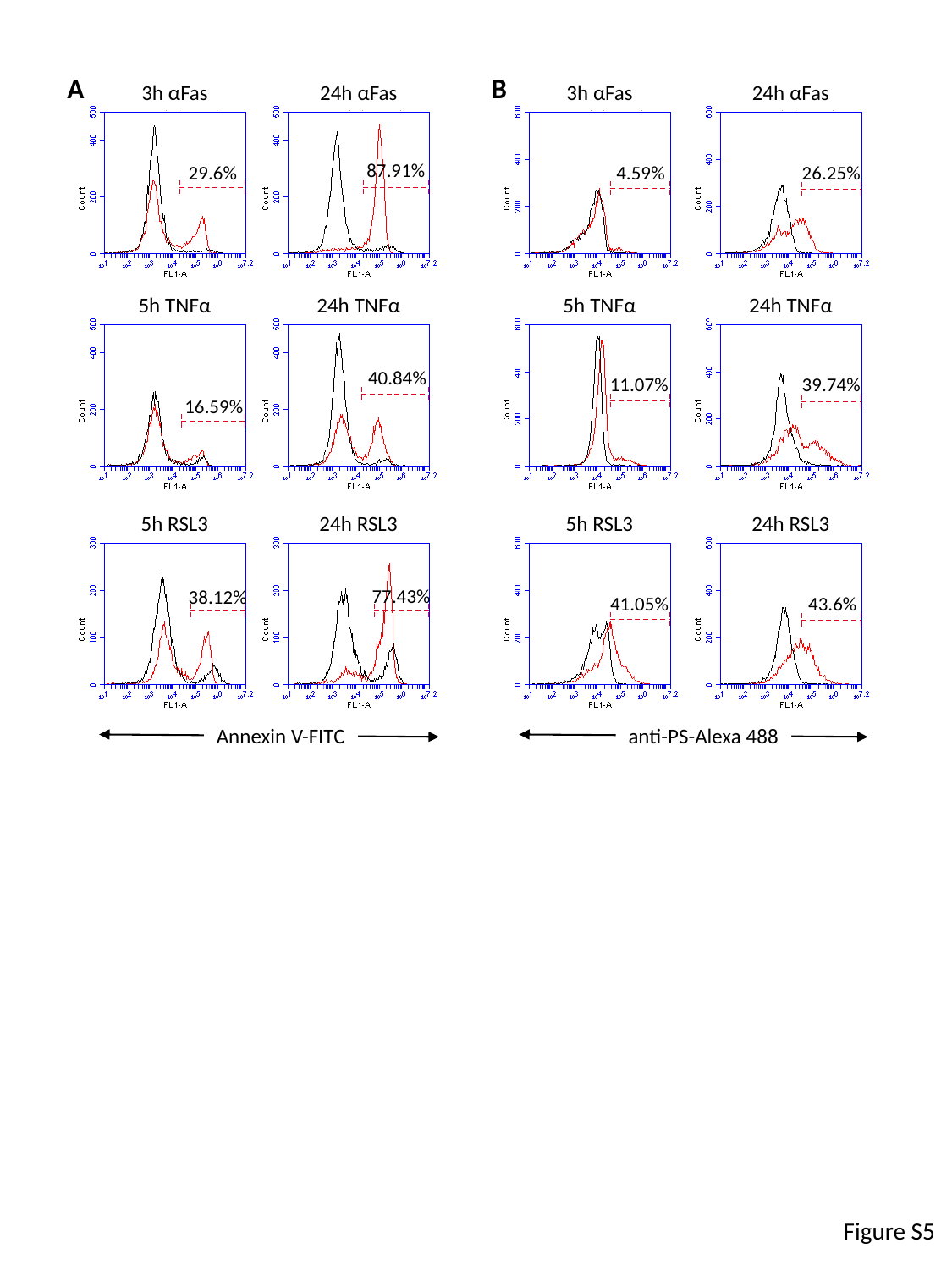

A
B
3h αFas
24h αFas
3h αFas
24h αFas
29.6%
4.59%
26.25%
87.91%
5h TNFα
24h TNFα
5h TNFα
24h TNFα
40.84%
11.07%
39.74%
16.59%
5h RSL3
24h RSL3
5h RSL3
24h RSL3
77.43%
38.12%
41.05%
43.6%
Annexin V-FITC
anti-PS-Alexa 488
Figure S5

## Slide 6
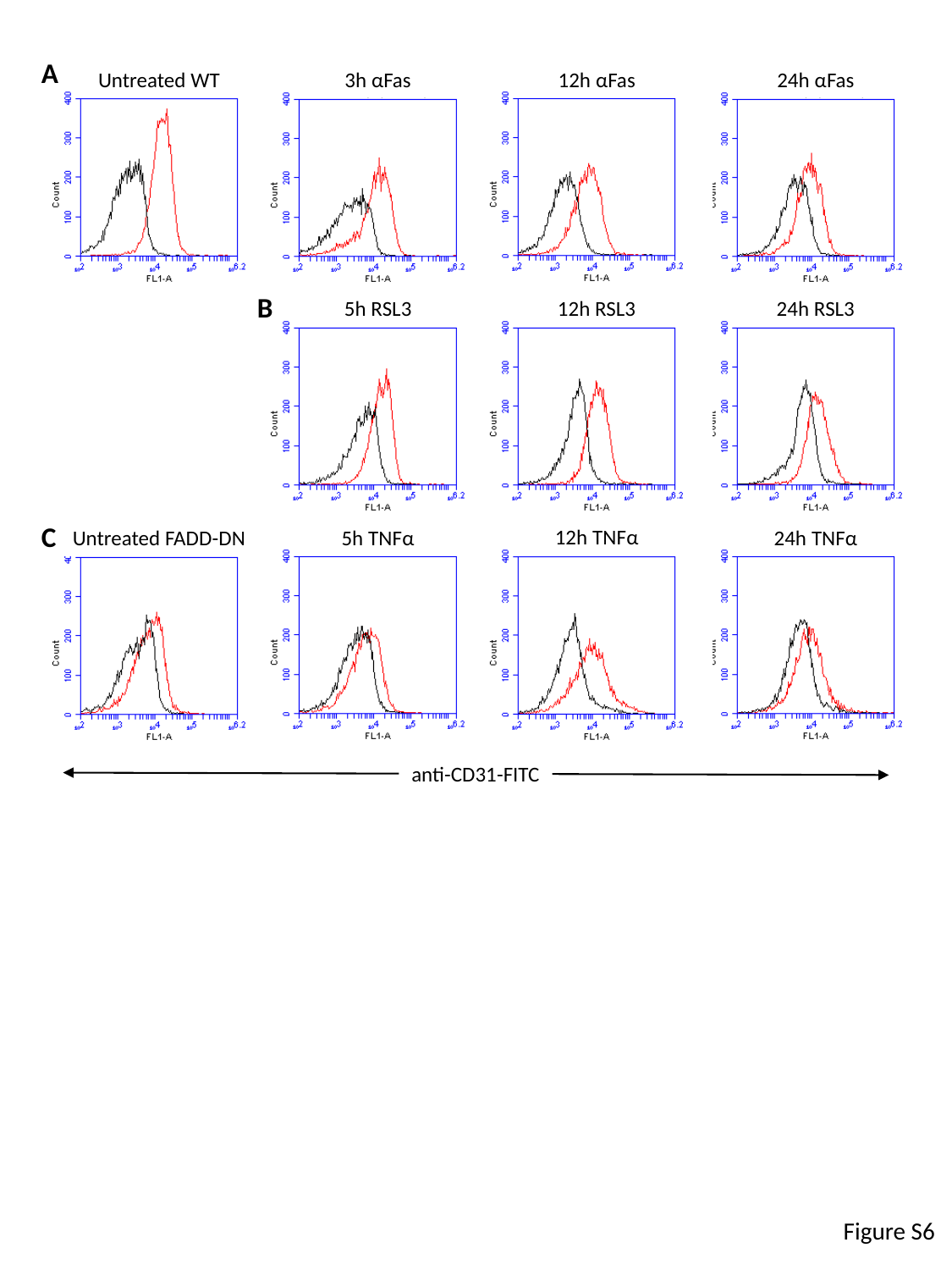

A
12h αFas
24h αFas
3h αFas
Untreated WT
B
5h RSL3
24h RSL3
12h RSL3
C
12h TNFα
5h TNFα
24h TNFα
Untreated FADD-DN
anti-CD31-FITC
Figure S6
