# Supplementary material for: Three cell deaths and a funeral: macrophage clearance of cells undergoing distinct modes of cell death
Source: Cell Death Discov. 2019 Feb 8;5:65. doi: 10.1038/s41420-019-0146-x (PMC6368547; doi:10.1038/s41420-019-0146-x)
Supplement: Supplementary file 2 — Supplemental Material File #1 [file 41420_2019_146_MOESM2_ESM.docx]

**Supporting figure legends**

Figure S1. Induction of apoptosis in Jurkat cells. TEM images of control cells (A, B), and cells triggered to undergo apoptosis with 250 ng/mL anti-Fas antibodies for 24 h (C, D). Note the presence of condensed chromatin upon Fas ligation. (E) Caspase 3-like activity as determined by the measurement of DEVD-AMC cleavage in Jurkat cells exposed to 250 ng/mL Fas antibody for 3 h in the presence (green line) or absence (red line) of the pan-caspase inhibitor, zVAD-fmk (10 µM), *versus* control cells (black line).

Figure S2. Induction of necroptosis in Jurkat FADD-DN cells. TEM images of control cells (A), and cells triggered to undergo necroptosis with 10 ng/mL TNF-α for 24 h (B-D). Note the presence of organelle swelling and lipid droplets. (E) Western blot showing a time-dependent increase in the expression of phosphorylated MLKL in cells treated with TNF-α, and its prevention by nec-1 (40 µM). The experiment was repeated three times.

Figure S3. Induction of ferroptosis in Jurkat cells. (A) Flow cytometric analysis of BODIPY^®^ 581/591-stained Jurkat cells exposed to 2 µM RSL3 (blue histogram). Lipid peroxidation was blocked by addition of 5 µM Ferrostatin-1 (green histogram). The black histogram represents untreated control cells. (B-D) TEM images of cells exposed to 2 µM RSL3 for 24 h. Note the alterations of nuclear morphology and lipid droplets.

Figure S4: Cell viability of apoptotic, necroptotic, and ferroptotic Jurkat or Jurkat FADD-DN cells. Apoptosis and ferroptosis were induced in Jurkat cells using 250 ng/mL Fas antibody or 2 µM RSL3, respectively. Necroptosis was induced in FADD-DN Jurkat cells using 10 ng/mL TNFα. After 24 h of cell death induction, cells were analyzed by flow cytometry. (A) The percentages of cells present in the gated population (refer to panel B) in relation to control samples are reported. Data are shown as relative values compared to the untreated control samples (n=3). (B) Representative FSC-SSC plots of the untreated and treated samples are shown. The gated population shows viable cells.

Figure S5. PS exposure in programmed cell death. Jurkat cells were triggered to undergo three different cell death modalities using anti-Fas antibodies (apoptosis), TNF-α (necroptosis), and RSL3 (ferroptosis), respectively, in the presence or absence of specific inhibitors. The cells were then stained using Annexin V-FITC (A) and anti-PS-Alexa 488 (B) to monitor PS exposure. In (A), red histograms: cells incubated with the cell death triggering agent, and black histograms are cells incubated with the cell death triggering agent plus the specific cell death inhibitor. In (B), red histograms represent samples stained with the PS-Alexa 488 antibody and black curves samples stained with the isotope-matched control antibody. The % PS-positive cells are shown.

Figure S6. CD31 expression. Three different cell death modalities were induced as indicated and cell surface expression of CD31 was investigated by using flow cytometry. Representative histograms of the CD31-FITC stained control *versus* apoptotic (A), ferroptotic (B), and necroptotic (C) cells are shown. The black line represents cells stained with the isotope control antibody and the red histogram represents cells stained with the CD31 antibody. Representative of three experiments.
